# Chromosome 9p terminal deletion in nine Egyptian patients and narrowing of the critical region for trigonocephaly

**DOI:** 10.1002/mgg3.1829

**Published:** 2021-10-05

**Authors:** Amal M. Mohamed, Alaa K. Kamel, Maha M. Eid, Ola M. Eid, Mona Mekkawy, Shymaa H. Hussein, Maha S. Zaki, Samira Esmail, Hanan H. Afifi, Ghada Y. El‐Kamah, Ghada A. Otaify, Heba Ahmed El‐Awady, Aya Elaidy, Mahmoud Y. Essa, Mona El‐Ruby, Engy A. Ashaat, Saida A. Hammad, Inas Mazen, Ghada M. H. Abdel‐Salam, Mona Aglan, Samia Temtamy

**Affiliations:** ^1^ Division of Human Genetics and Genome Research Department of Human Cytogenetics National Research Centre Cairo Egypt; ^2^ Division of Human Genetics and Genome Research Department of Clinical Genetics National Research Centre Cairo Egypt; ^3^ Department of Pediatrics Faculty of Medicine Fayoum University Fayoum Egypt

**Keywords:** 9p deletion, ambiguous genitalia, autism, brain anomalies, trigonocephaly

## Abstract

**Background:**

This study aimed to delineate the clinical phenotype of patients with 9p deletions, pinpoint the chromosomal breakpoints, and identify the critical region for trigonocephaly, which is a frequent finding in 9p terminal deletion.

**Methods:**

We investigated a cohort of nine patients with chromosome 9p terminal deletions who all displayed developmental delay, intellectual disability, hypotonia, and dysmorphic features. Of them, eight had trigonocephaly, seven had brain anomalies, seven had autistic manifestations, seven had fair hair, and six had a congenital heart defect (CHD).

**Results:**

Karyotyping revealed 9p terminal deletion in all patients, and patients 8 and 9 had additional duplication of other chromosomal segments. We used six bacterial artificial chromosome (BAC) clones that could identify the breakpoints at 17–20 Mb from the 9p terminus. Array CGH identified the precise extent of the deletion in six patients; the deleted regions ranged from 16 to 18.8 Mb in four patients, patient 8 had an 11.58 Mb deletion and patient 9 had a 2.3 Mb deletion.

**Conclusion:**

The gene deletion in the 9p24 region was insufficient to cause ambiguous genitalia because six of the nine patients had normal genitalia. We suggest that the critical region for trigonocephaly lies between 11,575 and 11,587 Mb from the chromosome 9p terminus. To the best of our knowledge, this is the minimal critical region reported for trigonocephaly in 9p deletion syndrome, and it warrants further delineation.

## Graphical abstract

Nine patients with chromosome 9p terminal deletion presented with developmental delay, intellectual disability, and dysmorphic features. We concluded that the deletion of the genes in the 9p24 region are not sufficient to cause ambiguous genitalia as six out of our nine patients had normal genitalia. Based on our study we suggested that the critical region for trigonocephaly may lies within 11.8 kb in 9p23 cytoband.

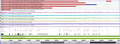

## 1 INTRODUCTION

9p deletion syndrome (Online Mendelian Inheritance in Man [OMIM]:# 158170) causes intellectual disability (ID) and developmental delay and is characterized by trigonocephaly and dysmorphic facial features in the form of malformed low‐set ears, upward‐slanting palpebral fissures, and midface hypoplasia. Reversed sex or impaired gonadal development has been observed in some patients. Alfi et al. (1973) were the first authors to describe 9p deletion syndrome. Many authors reported that *DMRT1* gene (OMIM# 602424), mapped to the 9p24.3 region, was responsible for the reversed sex (Barbaro et al., 2009; Ogata et al., 2001). Calvari et al. (2000) reported a patient with 46,XY reversed sex and a 9p terminal deletion extending 700 kb from the 9p terminus, with the breakpoint localized very close to, but outside of, *DMRT1* gene.

Many authors have attempted to identify the genes responsible for trigonocephaly, but they could only map this trait to a critical region located at 11−16Mb from the chromosome 9p terminus (Faas et al., 2007; Hauge et al., 2008; Mitsui et al., 2013; Shimojima & Yamamoto, 2009; Wagstaff & Hemann, 1995). Christ et al. (1999) studied 24 patients with 9p deletion syndrome and identified 10 different breakpoints, nine of which were in a region of ~5 Mb in chromosome 9p22−p23. Shimojima and Yamamoto (2009) postulated that certain genes, such as *CER1* (OMIM# 603777), *TYRP1* (OMIM# 115501), and *PTPRD* (OMIM# 601598), were responsible for the phenotype of 9p deletion syndrome. Many authors found that telomeric deletions were associated with gonadal dysgenesis (Calvari et al., 2000; Kawara et al., 2006; Onesimo et al., 2012; Swinkels et al., 2008), while proximal deletion caused a malformation syndrome (Christ et al., 1999; Wagstaff & Hemann, 1995). In their study of 10 patients with 9p deletion Hauge et al. (2008) mapped the region responsible for the disease between 11.8 and 16 Mb from the chromosome 9p terminus.

Many authors have reported the association between 9p deletion syndrome and the presence of autism spectrum disorders (ASD), some of whom reported that the genes located in the 9p24 region were responsible for ASD (Güneş et al., 2017; Vinci et al., 2007; Yang et al., 2012). *DOCK8* (OMIM# 611432), *CBWD1* (OMIM# 611078), *KANK1 (OMIM# 607704)*, and *FOXD4* (OMIM# 601092) genes are responsible for autistic behavior and speech and language disorders. These genes lie distal to *DMRT1* gene, (Lai et al., 2001; Ottolenghi et al., 2000; Ruusala & Aspenstrom, 2004). Here, we present nine patients with 9p terminal deletions with the aim of delineating the clinical presentation of patients with 9p deletions, identifying the chromosomal breakpoints, and defining the minimal critical region for trigonocephaly.

## 2 PATIENTS AND METHODS

### 2.1 Clinical summary

**FIGURE 1:**
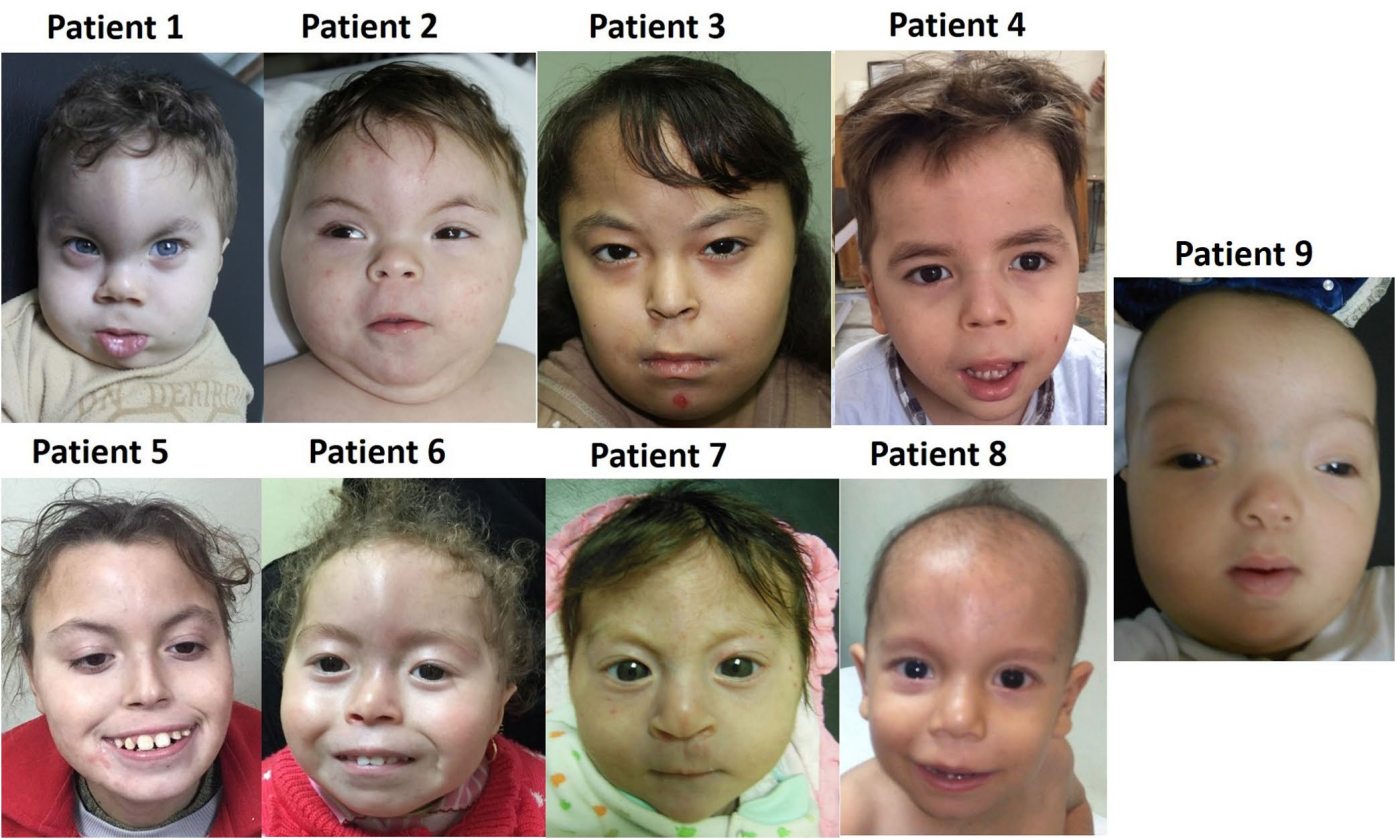
Facial characteristic of the nine patients with 9p deletions. Faces of patients 1−8, showing trigonocephaly, arched bushy eyebrows, upward‐slanting palpebral fissures, epicanthic folds, hypertelorism, upturned nostrils, a long philtrum, microstomia, and thin lips except for patient 1 who has a thick everted lower lip. Face of patient 9 showing a high forehead, frontal bossing, thin arched eyebrows, hypertelorism, a depressed nasal bridge, microstomia, a flat philtrum, and micrognathia

The study cohort comprised nine patients from nine unrelated Egyptian families, including six females and three males aged 3 months to 9 years. They were referred to our genetic clinics at the National Research Centre because of developmental delay and an abnormal skull shape. All had unremarkable pregnancy and delivery histories. Details of their clinical features are presented in Table 1. Eight patients had the characteristic skull shape of trigonocephaly and a prominent metopic suture, while one patient had a high and broad forehead and frontal bossing. All had a flat occiput and moderate to severe intellectual disability (ID). Moreover, six of the nine patients had associated autistic features. The characteristic facial dysmorphism of 9p deletion syndrome was present in all patients, including arched bushy eyebrows, hypertelorism, midface hypoplasia, a flat nasal bridge, anteverted nares, a long philtrum, a thin upper lip, and low‐set, posteriorly positioned ears (Figure 1). Moreover, they all had a short neck, widely spaced nipples, and hypotonia. Three patients had nystagmus. Hernias were present in four patients (two with inguinal hernias, one with an umbilical hernia, and one with both umbilical and inguinal hernias). Congenital heart defects (CHDs) were noted in six patients and included patent ductus arteriosus (PDA) in two patients, and atrial septal defect, both atrial septal defect and PDA, ventricular septal defect (VSD), and coarctation of the aorta in one patient each, respectively. Abnormal external genitalia were observed in three patients (two females had an absent clitoris and hypoplastic labia minora, and one male had hypospadias and a hypoplastic scrotum); however, none of our patients had reversed sex. All patients had long tapered fingers and flat narrow feet, and two also had misaligned and overlapping toes. Scoliosis was evident in one patient, and another had kyphosis. Anthropometric measurements revealed normal height and weight, except for patient 8, who was underweight. Microcephaly was noted in three of the nine patients. Brain magnetic resonance imaging (MRI) records were available for seven patients, revealing a hypoplastic corpus callosum in five and mild cortical atrophy in four patients. Fair hair was noted in seven patients. A history of repeated infections was noted in eight, constipation was reported in seven, and gastrointestinal reflux was found in five patients. Additionally, two patients experienced sleep disturbances.

**TABLE 1:** Clinical characteristics of the studied patients with 9P deletion in comparison with some other authors

	P 1	P 2	P 3	P 4	P 5	P 6	P 7	P 8	P 9	P No.	Swinkels et al. (2008) *p* = 13	Hauge et al. (2008) *p* = 10	Huret et al. (1988) *p* = 88
Age Y:M	0:5	0:11	9:00	3:00	6:00	1:10	0:03	2:08	0:09				
Sex	M	F	F	M	F	F	F	M	F				
Craniofacial features													
Trigonocephaly/prominent metopic suture	+	+	+	+	+	+	+	+	−	8/9	8/13	3/8	32/32
Flat occiput	+	+	+	+	+	+	+	+	+	9/9			
Choanal atresia	−	−	−	−	−	−	−	−	−	0/9			
Upslanting palpebral fissures	+	−	+	−	+	+	+	−	−	5/9	4/13		
Arched eye brows/bushy	+	+	+	+	+	+	+	+	+	9/9	5/13	2/6	9/12
Hypertelorism	+	+	+	+	+	+	−	+	+	8/9			
Epicanthal folds	+	+	−	+	+	+	+	+	+	8/9			
Nystagmus	−	−	−	+	+	+	−	−	−	3/9			
Midface hypoplasia	+	−	−	+	+	+	+	+	+	7/9	11/13	4/6	6/7
Flat nasal bridge	+	+	Slightly	+	+	+	+	+	+	9/9	8/13		27/28
Anteverted nares	+	+	Slightly	+	+	+	+	+	Slightly	9/9	9/13		30/32
Long flat philtrum	+	+	+	+	+	+	+	+	+	9/9	10/13	6/7	32/32
Low‐set, posteriorly positioned ears	+	+	+	+	+	+	+	+	+	9/9	9/13	6/9	27/32
Abnormal mandible	Micro‐gnathia				Prom‐inent mandible	Promi‐nent mandible		Micrognathia		4/9			
Microstomia	+	+	−	+	+	+	+	−	+	7/9			
Short neck	+	+	+	+	+	+	+	+	+	9/9			26/27
Globalized hypotonia	+	+	+	+	+	+	+	+	+	9/9	11/13		12/22
Widely spaced nipples	+	+	+	+	+	+	+	+	+	9/9	9/10	4/7	31/31
Cardiac defects	PDA	−	−	PDA	ASD	VSD	(ASD and PDA)	Coarctation of the aorta	NA	6/8	7/13	2/4	16/35
Inguinal Hernias Umbilical hernia	+ −	− −	+ +	+ −	−	− +	− −	− −	− −	3/9 2/9			16/35 −
Abnormal genitalia	−	Absent clitoris and labia minora	−	−	−	−	−	+ Hypospadias & Hypoplastic scrotum	Absent clitoris and labia minora	3/9	3/13	4/6	15/36
Hypopigmentation of skin/hair	+ Fair hair	+ Fair hair	−	+ Fair hair	+	+	−	+ Fair hair Sparse	Sparse hair	7/9			
Joint hyperlaxity	−	+	−	+	+	+	+	+	+	7/9	6/10		
Skeletal anomalies													
Long tapering fingers/toes	+	+	Toes mis‐aligned	+	+	+	−	+ bilateral misaligned toes	NA	7/8	10/13		5/18
Clinodactyly	+	−	+	+	−	−	+	+	NA	5/8		3/9	
Narrow flat feet	+	+	−	+	+	+	+	+	NA	7/8	8/11		
Other anomalies of Hands & feet	Talipes	Fusi‐form taper‐ing fingers	Fusiform tapering fingers & mis‐aligned Toes	−	Widely spaced toes	−	Campto‐dactyly in hands, talipes and over‐lapping toes	Long big &2nd toes, bilateral short dysplastic 3rd toe and bilateral proximal thumb displacement.	NA				
Scoliosis	−	−	−	−	+	−	−	−	Kyphosis	2/9	5/13		9/21
Precocious puberty	−	−	−	−	−	−	−	−	−	0/9			
Seizures	−	−	−	−	+	−	−	−	−	1/9			
Gastrointestinal reflux	−	−	−	+	+	+	+	+	−	5/9			
Constipation	+	+	−	+	+	+	+	+	−	7/9			
Frequent infections	+	+	+	+	+	+	+	+	−	8/9	9/11		
Sleep disorders	−	−	−	−	+	+	−	+	−	3/9			
Excessive drooling	−	−	−	+	+	−	−	+	−	3/9			
Glaucoma/Cataract	−	−	−	−	−	−	−/−	−	−	0/9			
Developmental delay	+ DQ 48% Moderate	+ DQ 20% Severe	+ 50 (mild to moderate)	+ 50	+ Severe	+ Moderate	+ 40 Moderate	+ Severe	+ Severe	9/9	13/13		36/36
Autistic behavior	+	+	−	+	+	+	−	+	−	6/9			
Speech/language delay	Early to evaluate	+	+ Mild	+ Absent speech	+ Absent speech	+ Absent speech	Early to evaluate	+ Absent speech	Early to evaluate	6/6			
Anthropometry (SD)													
Weight	Mean	−0.5 SD	Mean	−1 SD	−1 SD	−1.5 SD	−1.8 SD	−2.9 SD	+2 SD	8/9 mean			
Height	−0.5 SD	Mean	−2 SD	−2 SD	−2.1 SD	−1 SD	−1.3 SD	Mean −0.4	+2 SD	9/9 mean	9/9 mean		
Head circumference	−0.5 SD	−2.9 SD	−3.1 SD	−1 SD	−2 SD	−3.2 SD	−0.9 SD	Mean	+2 SD	3/8 microcephaly			
Brain MRI findings	Hypo‐plastic corpus callosum	Lt occipitoparietal area of hypo‐density due to HIE.	NA	Mild cortic‐al and central atro‐phy	Mild cortical atrophy, hypo‐plastic corpus callosum and prominent Sylvian fissure	Hypo‐plastic corpus callosum	Hypo‐plastic frontal lobe and hypoplasti‐c corpus callosum	Mild cortical and central atrophy Hypoplastic corpus Callosum	NA	7/7		1/8	

Abbreviations: −, absent; +, present; ASD, atrial septal defect; DQ, developmental quotient; F, female; M, male; NA, not available; P, patient; PDA, patent ductus arteriosus; SD, standard deviationVSD, ventricular septal defect; Y:M, year:Month.

### 2.2 Methods

Cytogenetic analysis was performed on peripheral blood lymphocytes for the nine probands and their parents at the level of 550 cytobands. The nomenclature used was according to the International System for Human Cytogenomic Nomenclature 2016 (ISCN).

We used six BAC clones in four patients (patients 3, 5, 6, and 7): four spanning the 9p21−p23 region (test probes) and two encompassing 9q13 and 9q34.3 (control probes). We labeled the BAC clones via nick translation kit (Abbott), followed by precipitation of the probe DNA, lyophilization using SpeedVac centrifuge (Eppendorf) and dissolution of the probe in hybridization buffer for use in fluorescence in situ hybridization (FISH).

Array comparative genomic hybridization (CGH) was performed in patients 1, 2, 3, 5, 8, and 9 using CytoScan 750K and Cytoscan HD gene chips (Affymetrix). Hybridization of the gene chips was performed for 16 h using a GeneChip Hybridization Oven 645 (Affymetrix), followed by washing in a Genechip Fluidics Station 450 (Affymetrix) and scanning on a GeneChip Scanner 3000 (Affymetrix). Data analysis was performed using Chromosome Analysis Suite software (Affymetrix).

## 3 RESULTS

### 3.1 Karyotype results

**FIGURE 2:**
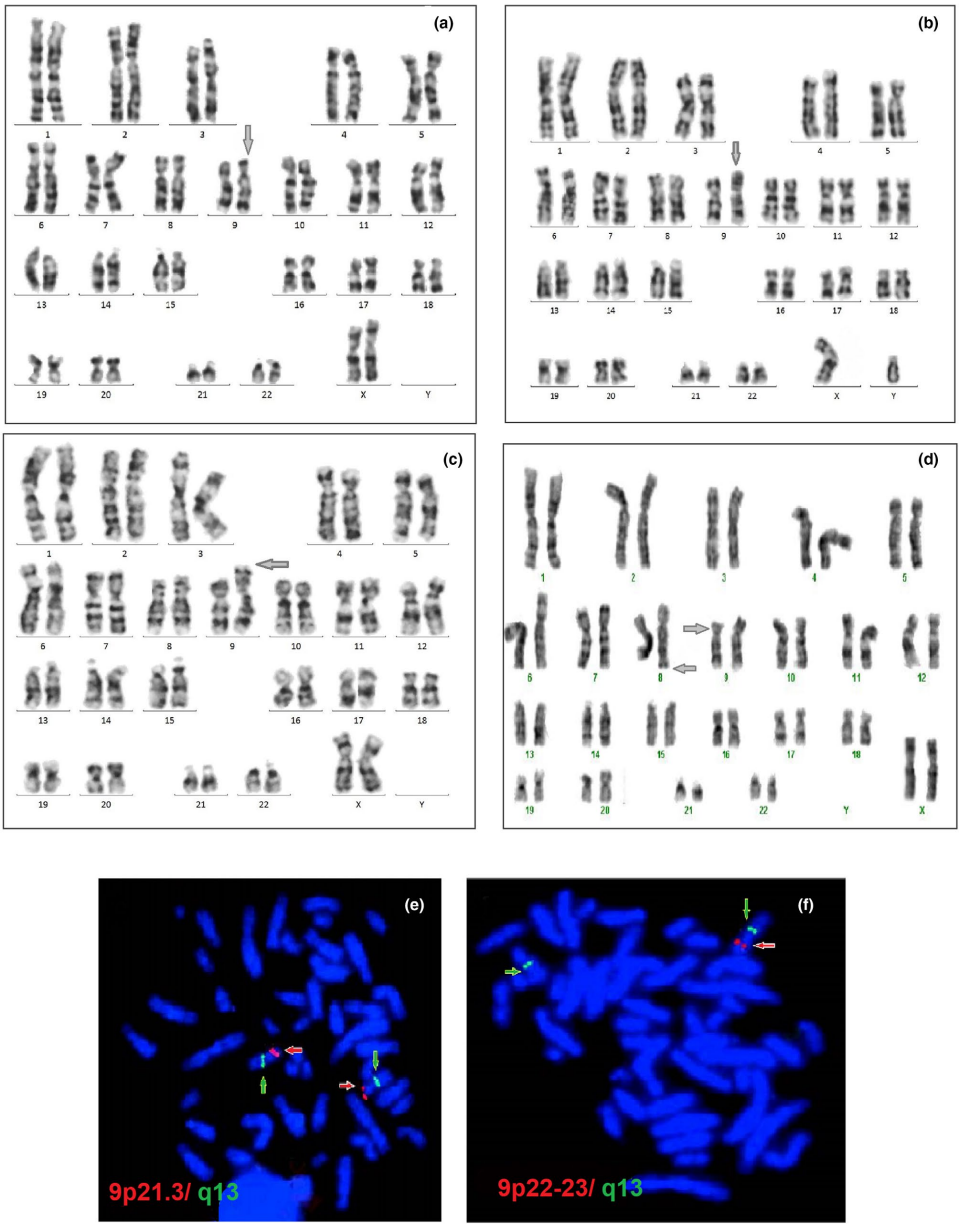
Karyotyping results of the patients with 9p deletions. (a) Karyotype 46,XX,del(9)(p22), this is an example for of 9p terminal deletion because there is no difference at the karyotype level in the extent of the deletion in the seven patients with 9p terminal deletion. (b) Karyotype of patient 8 with 46,XY,der(9)t(8;9)(p21;p23). (c) Karyotype of patient 9 show 46,XX,der(9)t(7;9)(p15;p24). (d) Karyotype of the mother of patient 7 with 46,XX,t(8;9)(q24.3;P21.1). (e) BAC FISH results of patient 6. Red arrow, RP11‐730N17 BAC FISH probe (used as the test probe at 9p21.3) was not deleted. Green arrow, RP11‐370H5 BAC FISH probe (used as the control probe at 9q13). (f) Red arrow, RP11‐87N24 BAC FISH probe (used as the test probe at 9p22−23) was deleted. Green arrow, RP11‐370H5 BAC FISH probe (used as the control probe at 9q13)

Patients 1−7 had pure 9p terminal deletion (see Figure 2a as an example of del(9)(p22)), patient 8 had a 46,XY,der(9)t(8;9)(p21;p23) karyotype (Figure 2b), and patient 9 had a 46,XX,der(9)t(7;9)(p15;p24) karyotype (Figure 2c). All parents had a normal karyotype, with the exception of the mother of patient 7, who had a 46,XX,t(8;9)(q24.3;P21.1) balanced translocation, (Figure 2d).

### 3.2 BAC Clones

BAC clones were used in four patients (patients 3, 5, 6, and 7). We used six BAC clones, four spanning the distal 9p23−p21 region (test probes), and two (9q13 or 9q34.3) serving as controls.

The proximal RP11‐730N17 and RP11‐175M8 probes on 9p21.3 were not deleted. The RP11‐163F8 and RP 11‐87N24 probes on 9p22 and 9p22–p23, respectively, were deleted (Figure 2e,f). These results indicated that the breakpoints lay between the RP11‐163F8 and RP11‐175M8 probes, which are located in a region between 17,770 and 20,602 Mb from the 9p terminus. Figure 2e,f depicts the deleted and non‐deleted BAC clones, and Table 2 shows the deleted and non‐deleted BAC clones on chromosome 9p.

**TABLE 2:** BAC clones on chromosome 9p

BAC clone	Start	End	Cytoband	Patient 3	Patient 5	Patient 6	Patient 7	
RP11‐730N17	23,700949	23,808886	9p21.3	Not deleted	Not deleted	+ Not deleted	+ Not deleted	Not deleted in all
RP11‐175M8	20,453815	20,601848	9p21.3	Not deleted	Not deleted	Not deleted	Not deleted	Not deleted in all
RP11‐163F8	17,380K	17,770K	9p22	Deleted	Deleted	Deleted	Deleted	Deleted in all
RP 11‐87N24	10,350k	10,780k	9p22‐23	Deleted	Deleted	Deleted	Deleted	Deleted in all
RP11‐141J	70,572K	70,944K	9q13			Control	Control	Control
RP11‐370H5	139299023	139468177	9q34.3 Control	Control	Control			Control

### 3.3 Array CGH

Array CGH was performed in six patients (patients 1, 2, 3, 5, 8, and 9). In patients 1, 2, 3, and 5, the deleted regions in 9p ranged from 16.8 to 18.9 Mb. Patient 1 had an extra deleted segment of 1.1 Mb at 9p22.3, whereas patient 3 had a 2.47 Mb duplicated segment at 9p22.3−p22.1. Patient 8 had an 11.5 Mb deletion in the 9p terminus and 19 Mb duplication in chromosome 8p. Patient 9 had a 2.3 Mb deletion in the 9p terminus and a 26.8 = Mb duplication of the 7p terminus.

**FIGURE 3:**
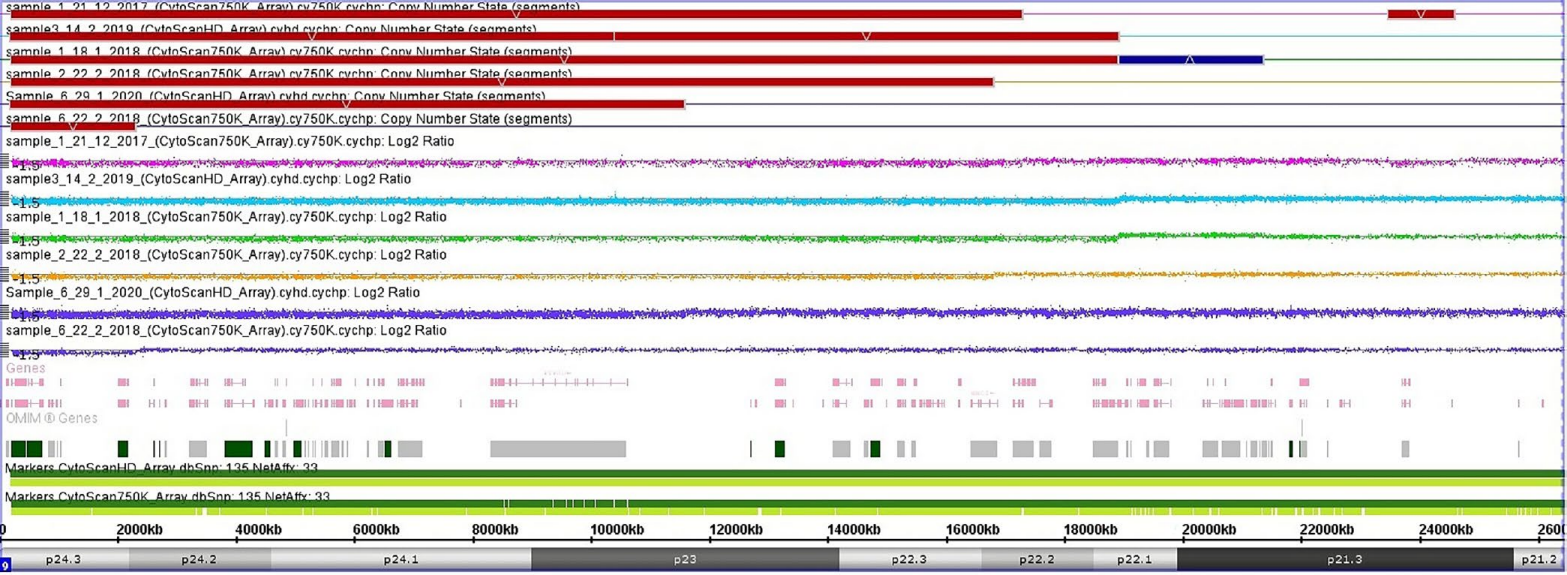
Array CGH of chromosome 9 for patients 1, 2, 3, 5, 8, and 9, showing the copy number of 9p, the horizontal red bars indicate the extent of the deleted segments in the six patients, and the blue bar indicates duplication. The Log2 ratio indicates the microdeletions and microduplication where the normal Log2 ratio = 0, deletion = −0.45, in duplication = 0.3

Figure 3 shows the Array CGH results of patients 1, 2, 3, 5, 8, and 9, with deleted 9p and extra deleted and duplicated 9p segments. Table 3 shows the array CGH results and the extent of the deleted 9p terminus as well as the additional deleted and duplicated segments (genome build GRCh37).

**TABLE 3:** Array CGH results for 9p deletion and extra deleted and duplicated regions

Patient 1	Patient 2	Patient 3	Patient 5	Patient 8	Patient 9
9p24.3p22.2	9p24.3p22.1	9p24.3p22.1	9p24.3p22.2	9p24.3p23	9p24,3p24.2
17.7 Mb	18.9 Mb	18.8 Mb	16.8 Mb	11.587 Mb	2.3 Mb
arr[GRCh37]9p24.3p22.1(208454_17724146)x1	arr[GRCh37]9p24.3p22.1(203861_18921363)x1	arr[GRCh37]9p24.3p22.1(208454_18893255)x1	arr[GRCh37]9p24.3p22.2(208454_16880694)x1	arr[GRCh37]9p24.3p23(203861_11587302)x1	arr[GRCh37]9p24.3p24.2(208454_2303882)x1
63 OMIM genes	65 OMIM genes	65 OMIM genes	61 OMIM genes	48 OMIM genes	15 OMIM genes
9p21.3		9p22.1p22.3		8p23.3p21.3	7p22.3p15.2
1.1 Mb (del)		2.47 Mb (dup)		19.4 Mb (dup)	26.8 Mb (dup)
arr[GRCh37]9p21.3(23480773_24598119)x1		arr[GRCh37]9p22.19p21.3(18894506_21369319)x3		arr[GRCh37]8p23.3p21.3(208048_19434723)x3	arr[GRCh37]7p22.3p15.2(43376_26843542)x3
16 OMIM genes		42 OMIM genes		89 OMIM genes	121 OMIM genes

**FIGURE 4:**
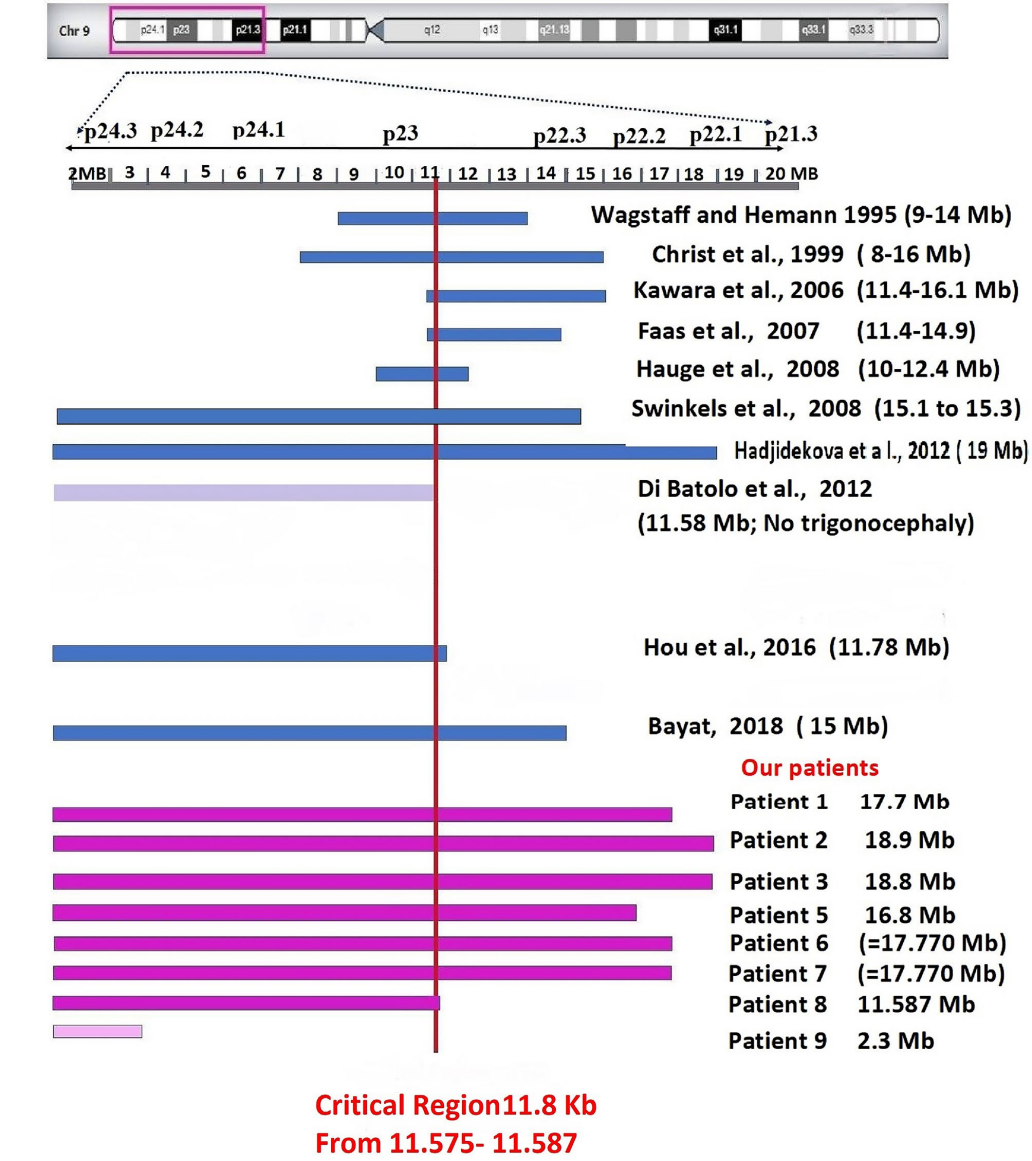
Diagram comparing the deleted region in our patients with those reported by other authors to identify the minimal critical region for trigonocephaly. The first horizontal line shows the 9p24p21 cytobands, and the second horizontal line represents the length of 9p in Mb. The blue horizontal lines represent the extent of deleted 9p segments in patients with trigonocephaly, as reported by other authors. The horizontal pink lines represent the extent of the deleted 9p segment in our patients with trigonocephaly. The grey horizontal line represents the extent of deleted 9p with no trigonocephaly. The light pink horizontal line represents our patient 9, with a 2.3 Mb deletion and no trigonocephaly. The vertical red line indicates the suggested critical region that encompasses our patients and other authors’ patients who have trigonocephaly

Figure 4 compares the 9p terminal deletion regions in our patients with those reported by other authors to identify the minimal critical region for trigonocephaly.

## 4 DISCUSSION

9p deletion syndrome is a multiple congenital anomaly/developmental delay syndrome characterized by cardinal manifestations of dysmorphic facial features, male sex reversal, trigonocephaly, and multiple congenital anomalies. There is an association between the distal 9p (9p24.3) deletion and autism, ID, delayed speech, behavioral problems, 46,XY reversed sex (female external genitalia), and ambiguous genitalia in both sexes. Deletion of the proximal region (9p23−p22) is responsible for the clinical manifestations of 9p deletion syndrome specifically trigonocephaly (Hauge et al., 2008; Swinkels et al., 2008).

All our patients presented with the main clinical manifestations of 9p deletion syndrome including trigonocephaly, except for patient 9, who had a 2.3 Mb 9p terminal deletion and presented with abnormal genitalia. In patients 1−8, the deletion ranged from 11.58 to 18 Mb.

Behavioral abnormalities, ID, delayed speech, and autistic behavior are common features of 9p deletion syndrome. In our study, we reported seven patients who had autistic manifestations. This could be attributed to deletion of *FOXD4* gene. *DOCK8*, *KANK1*, and *FOXD4* are located distal to *DMRT1* gene and their deletion causes behavioral abnormalities, ID, delayed speech, and autistic behavior. Hauge et al. (2008) reported 10 patients with 9p deletion, all of whom had autistic‐ like behavior and behavioral problems, delayed speech, and language impairment in all above 1‐year‐old patients. They all shared a minimal deleted region at 9p24.3 of ~800 kb that involved *FOXD4* gene. Eight of our patients had recurrent infections, which may be attributed to *DOCK8* gene deletion.

Brain anomalies are observed in almost all patients with 9p deletion. In our study, seven patients had abnormal brain computed tomography (CT), (CT results were unavailable for patients 6 and 9), five showed varying degrees of corpus callosum malformation in neuroimaging findings, ranging from complete agenesis to isolated hypogenesis of the corpus callosum, or association with other cortical malformations in the form of mild cortical atrophy.

In 2018, Schanze et al., described 18 patients with macrocephaly, acquired, with impaired intellectual development (MACID) syndrome, in which some patients had corpus callosum abnormalities. Of them, 11 had deletions ranging from 225 kb to 4.2 MB on 9p23−p22, where the shortest region included *NFIB* gene (OMIM# 600728) alone.

Although reversed sex is another feature of male patients with 9p deletion, this was not observed in any of our male patients. However, three of our patients had abnormal genitalia; two females and one male. Patient 2 with an 18.9 Mb terminal deletion exhibited an absent clitoris and hypoplastic labia minora, while patient 8, a male with an 11.58 Mb 9p terminal deletion, exhibited hypospadias. Patient 9 had a 2.3 Mb 9p terminal deletion and abnormal genitalia. These data support the hypothesis that abnormal genitalia are related to 9p24.3 terminal deletion encompassing the *DMRT* gene family.

Guioli et al. (1998) described five 46,XY males who had deletion of 9p24; two had female external genitalia and three had ambiguous genitalia. They concluded that 9p terminus was critical for male sex development.

Huret et al. (1988) evaluated 80 patients with 9p deletion, of whom 42% had external genital anomalies. The 46,XX individuals did exhibit gonadal dysgenesis and had less ambiguous genitalia. Moreover, Quinonez et al. (2013) reviewed 88 patients with a 46,XY karyotype who had 9p deletion, of whom 60% had gonadal dysgenesis or ambiguous genitalia. They concluded that the hemizygous deletion of 9p24.3 (including *DMRT* gene), was insufficient to cause sex reversal or ambiguous genitalia in many patients with 9p terminal deletion. This could be explained by additional genes on other chromosomes being responsible for the development of genital organs that are important in both males and females for gonadogenesis and sex determination (Hauge et al., 2008).

The most common CHD defects reported in 9p deletion syndrome are VSD, atrial septal defect, and PDA (Pierpont et al., 2018). *KDM4C* (OMIM# 605469) on 9p24.1 is responsible for cardiac differentiation in murine embryos, whereas *CER1* located on 9p22.3 is associated with CHD. In turn, mutation of *FOXD4* on 9p24.3 is associated with dilated cardiomyopathy (Pierpont et al., 2018). CHD was present in six of our nine patients. Coarctation of the aorta is a rare manifestation of 9p deletion syndrome, which was reported by Koslow et al. (2018) and observed in our patient 8. Other rare manifestations may be associated with 9p deletion syndrome like neonatal hyperinsulinemia/hypoglycemia (Bayata et al., 2018) and arthrogryposis multiplex congenita (Kim et al., 2017).

Some authors have reported patients with 9p deletion associated with other chromosomal abnormalities. Our patient 8 had a derivative chromosome 9 with an 11.5 Mb 9p terminal deletion and duplication of 19 Mb of 8p23.3−p21.3, karyotype 46,XY,der(9)t(8;9)(p21;p23). 8p duplication syndrome is characterized by mild or moderate developmental delays, mild dysmorphism, CHD, and behavioral disorders (Barber et al., 2013). Our patient 8 had the main characteristic features of 9p deletion syndrome rather than those of 8p duplication syndrome.

Moreover, our patient 9 had a karyotype of 46,XX,der(9)t(7;9)(p15;p24), with the deletion of 2.3 Mb of 9p224.2 and duplication of 26.8 Mb of 7p22.3−p15.2. This patient presented with dysmorphic features and abnormal genitalia in the form of an absent clitoris and hypoplastic labia minora, but no trigonocephaly. These features were more suggestive of 7p duplication syndrome, which includes ID, skeletal abnormalities, CHD, and dysmorphic features in the form of large anterior fontanel, a broad forehead (observed in our patient 9), hypertelorism, and low‐set ears (Cox and Butler, (2015; Goitia et al., 2015; Papadopoulou et al., 2006).

Many studies have been performed to define the critical region on chromosome 9p that is responsible for the clinical presentation of 9p deletion syndrome. Christ et al. (1999) performed cytogenetic and molecular analysis to identify breakpoint cluster regions in 24 patients who had 9p deletion and narrowed the critical region as extended for 8 Mb from marker D9S285 to marker D9S286, encompassing bands 9p23−p22 (8−16 Mb), starting from the 9p terminus. Moreover, Hauge et al. (2008) reported 10 patients with 9p deletions of varying sizes. None of them had reversed sex, but four had ambiguous genitalia, five had deletions <7 Mb, and four had deletions of 10−12.4 Mb. However, trigonocephaly was only present in three patients; one with a 2 Mb terminal deletion of 9p and the other two with a deletion of 10−12.4 Mb. Hauge et al. (2008) suggested 9p22.3 as the hotspot of 9p deletion syndrome. Swinkels et al. (2008) analyzed 13 Dutch patients with 9p deletion, in whom 50% had the clinical picture of 9p deletion syndrome; three of five males had disorders of sex development, seven patients had CHD, and two had ASD. The authors concluded that the critical region responsible for 9p deletion syndrome lay within a 300 Kb region on 9p22.3. Abreu et al. (2014) reported two cousins who were born to identical twin sisters and had a deletion in the 9p24.3 region associated with the phenotype of 9p deletion syndrome. In that study, although FISH and multiplex ligation‐dependent probe amplification (MLPA) techniques with probes that covered only copy number variants at the telomeric regions, were used, the authors postulated that the deletions were only located at these sites. However, they did not use probes or markers for the detection of deletions extending beyond the 9p23 or p22 loci. The presence of the 9p‐deletion phenotype in these two reported patients indicates that the deletions may extended to more proximal regions. This highlights the importance of array CGH to detect the exact extension of deleted and duplicated segments.

Spazzapan et al. (2016) reported six patients with the clinical picture of 9p deletion syndrome who all had trigonocephaly and abnormal brain CT findings, with an abnormal corpus callosum only observed in four patients. All had a deletion involving the 9p23−p22 region. Several authors have narrowed down the critical region of trigonocephaly to a 3.5 Mb segment extending from 11.4 to 14.9 Mb from the 9p terminus (Christ et al., 1999; Huret et al., 1988; Kawara et al., 2006).

Although *CER1* and *FREM1* (OMIM# 608944) were suggested as responsible for trigonocephaly, in our patient 8, who had trigonocephaly, the *CER1* and *FREM1* loci were not part of the deleted 9p region. This suggests that *CER1* and *FRM1* are not the only genes responsible for trigonocephaly and that other distal genes may be responsible. This finding is supported by the results of Swinkels et al. (2008), who did not find *CER1* deletions or mutations in sequencing data of nine patients with isolated trigonocephaly.

In our patient 8, the 9p deletion extended 11.58 Mb from the 9p terminus to 9p23. This patient had the clinical picture of 9p deletion syndrome including trigonocephaly, suggesting that the critical region for the clinical picture of 9p deletion lies within the 9p23 segment, which is supported by the findings of Hauge et al. (2008) and Hou et al. (2016). In the patient reported by Recalcati et al. (2012) and patient 9 of Swinkels et al. (2008), the absence of trigonocephaly in the presence of a deletion >15 Mb may be due to reduced penetrance. Although Swinkels et al. (2008) characterized the critical region of trigonocephaly as a 300 kb segment within 15.1−15.3 Mb from the 9p terminus (9p22.3), according to the range of interstitial deletions in their patients 1−3, the authors did not use BAC clones that covered the region from 8.3 to 14 Mb in these patients. Therefore, they defined a more proximal region as being critical. The use of array CGH in their patients 6−9 allowed these authors to define the exact range of the deleted regions. This indicates the importance of array CGH for the precise identification of the size of deletions and duplications.

Our findings agree with Christ et al. (1999), who reported a deletion of 8–16 Mb, starting from the 9p terminus. Moreover, they were in accordance with those of Hauge et al. (2008), who reported two patients with trigonocephaly and a deletion of 10−12.4 Mb.

Di Bartolo et al. (2012) described a female patient with an 11.57 Mb deletion of 9p24.3−p23 and a duplication of 32.7 Mb from 9p23 to 9p11.2. The patient exhibited clinical manifestations of 9p trisomy and some of 9p deletion; she did not have trigonocephaly and had CHD and normal genitalia. In contrast to trigonocephaly, she had delayed closure of the anterior fontanelle and sagittal suture, which are manifestations of 9p duplication. The patient reported by Di Bartolo et al. (2012) had the 9p deletion breakpoint at 11,575,785 and no trigonocephaly, and our patient 8 had the breakpoint at 11,587,02 and had trigonocephaly. This suggested that the selected critical region for trigonocephaly lies within 11.8 kb in 9p23 (11,575,785 to 11,587,302). Thus, we propose that this segment responsible for trigonocephaly. Although there are no reported genes in this segment, OMIM defines eight phenotype loci related to this segment. The region is flanked by two uncharacterized transcripts that may be essential for transcriptional regulation of other genes related to 9p deletion syndrome including trigonocephaly. https://www.ncbi.nlm.nih.gov/nucleotide/XR_929476.2?report=genbank&log$=nuclalign&blast_rank=1&RID=AAUBVF8W01N. https://www.ncbi.nlm.nih.gov/nucleotide/XR_929479.1?report=genbank&log$=nuclalign&blast_rank=4&RID=AAUBVF8W01N.

We suggest that this 11.8 kb region in 9p23, is the most critical region for trigonocephaly. Further research is necessary to detect mutations in this region, especially in patients with isolated trigonocephaly.

Our suggested critical region is in accordance with several reports of deletions that encompass our region of trigonocephaly (Christ et al., 1999; Faas et al., 2007; Hauge et al., 2008; Kawara et al., 2006; Mitsui et al., 2013; Shimojima & Yamamoto, 2009; Swinkels et al., 2008; Wagstaff & Hemann, 1995). There are very few exceptions to our suggested critical region, Mitsui et al. (2013) reported a patient with a 9 Mb deletion from the 9p terminus, and Hauge et al. (2008) described 9p terminal deletion of 4 Mb in patient no. 4, both of whom had trigonocephaly. The variance between different authors in the suggested critical region for 9p deletion syndrome including trigonocephaly may indicate that there is more than one gene on 9p and that its deletion may contribute to trigonocephaly. Furthermore, our suggested critical region may contain a regulatory sequence essential for the transcription of one or more of these genes.

Figure 4 represents the deleted 9p regions in our patients and those reported by other authors to identify the minimal critical region for trigonocephaly. Most of the reported critical regions for trigonocephaly encompassed our suggested critical region (with few exceptions). Several authors have narrowed down the critical region of trigonocephaly to a 3.5 Mb segment extending from 11.4 to 14.9 Mb from the 9p terminus, and the critical region for the clinical picture of 9p deletion lies within the 9p23 segment. The region of Di Bartolo et al. (2012) was 11,575 Mb in a patient with no trigonocephaly, that of Hou et al. (2016) was 11.8 Mb in a patient with trigonocephaly and that of our patient 8, who had trigonocephaly was 11,587 Mb. These data are the most supportive of our suggested critical region (11,575−1,587 Mb). We suggest further study to detect mutations in this region, particularly in patients with isolated trigonocephaly.

## 5 CONCLUSIONS

In conclusion, the clinical findings of our cohort (except patient 9) reflect the typical phenotype of 9p deletion syndrome. Hypopigmentation of hair and skin was an additional feature in most of our patients and has not been previously reported. The phenotypic features and the range of deleted regions led us to conclude that the distal 2.3 Mb 9p terminal region carries genes that are responsible for gonadogenesis in both females and males. Moreover, a breakpoint cluster region was identified in the segment through 16−18 Mb, which was present in six of our patients and has been reported by other authors. The translocation (8;9) is not random and a palindromic region may be involved between 8p and 9p because this is a recurrent locus for translocation. In conclusion, we suggest that the genome location of 11,587,302−11,575,785 in band 9p23 is the critical region for trigonocephaly. To the best of our knowledge, this is the minimal critical region reported for trigonocephaly, and it warrants further delineation.

## CONFLICT OF INTEREST

The authors have declared no conflicts of interest.

## AUTHOR CONTRIBUTIONS

A.M. planned the study, wrote the manuscript, coordinated between the authors, and revised and edited the manuscript. A.K., M.M., M.E., and S.H. performed the cytogenetic data analysis, reviewed the manuscript, performed the BAC analysis, and contributed toward manuscript revision. O.E. and S.H. performed the microarray analysis and interpretation. M.Z., S. E., H.A., G. E., M.R., I. M., G.A., and M.A. provided clinical data, performed patients’ clinical evaluation, and contributed toward manuscript revision. G.O., H.E., A.E., M.E., and E.A. performed the clinical evaluation and wrote the clinical report, patients’ referral, evaluation of the brain anomalies, and developmental evaluation. S.T. performed the final revision and editing of the manuscript.

## Data Availability

The data supporting the findings of this study are available from the corresponding author upon request.

## References

[mgg31829-bib-0001] Abreu, L. S. , Brassesco, M. S. , Moreira, M. L. C. , & Pina‐Neto, J. M. (2014). Familial balanced translocation leading to an offspring with phenotypic manifestations of 9p syndrome. Genetics and Molecular Research, 13, 4302–4310. 10.4238/2014.June.9.16 25036174

[mgg31829-bib-0002] Alfi, O. , Donnell, G. N. , Crandall, B. F. , Derencsenyi, A. , & Menon, R. (1973). Deletion of the short arm of chromosome no.9 (46,9p‐): Anew deletion syndrome. Annales De Genetique, 16, 17–22. 4541805

[mgg31829-bib-0003] Barbaro, M. , Balsamo, A. , Anderlid, B. M. , Myhre, A. G. , Gennari, M. , Nicoletti, A. , Pittalis, M. C. , Oscarson, M. , & Wedell, A. (2009). Characterization of deletions at 9p affecting the candidate regions for sex reversal and deletion 9p syndrome by MLPA. European Journal of Human Genetics, 17, 1439–1447. 10.1038/ejhg.2009.70 19417767 PMC2986678

[mgg31829-bib-0004] Barber, J. C. K. , Rosenfeld, J. A. , Foulds, N. , Laird, S. , Bateman, M. S. , Thomas, N. S. , Baker, S. , Maloney, V. K. , Anilkumar, A. , Smith, W. E. , Banks, V. , Ellingwood, S. , Kharbutli, Y. , Mehta, L. , Eddleman, K. A. , Marble, M. , Zambrano, R. , Crolla, J. A. , & Lamb, A. N. (2013). 8p23.1 Duplication syndrome; common, confirmed, and novel features in six further patients. American Journal of Medical Genetics. Part A, 161A, 487–500. 10.1002/ajmg.a.35767 23345203

[mgg31829-bib-0005] Bayata, A. , Kirchhoffb, M. , Madsenc, C. G. , & Kreiborgd, S. (2018). Neonatal hyperinsulinemic hypoglycemia in a patient with 9p deletion syndrome. European Journal of Medical Genetics, 61, 473–477. 10.1016/j.ejmg.2018.03.009 29601900

[mgg31829-bib-0006] Calvari, V. , Bertini, V. , De Grandi, A. , Peverali, G. , Zuffardi, O. , Ferguson‐Smith, M. , Knudtzon, J. , Camerino, G. , Borsani, G. , & Guioli, S. (2000). A new submicroscopic deletion that refines the 9p region for sex reversal. Genomics, 65, 203–212. 10.1006/geno.2000.6160 10857744

[mgg31829-bib-0007] Christ, L. A. , Crowe, C. A. , Micale, M. A. , Conroy, J. M. , & Schwartz, S. (1999). Chromosome breakage hotspots and delineation of the critical region for the 9p deletion syndrome. American Journal of Human Genetics, 65, 1387–1395. 10.1086/302606 10521304 PMC1288291

[mgg31829-bib-0008] Cox, D. M. , & Butler, M. G. (2015). A case of the 7p22.2 microduplication: Refinement of the critical chromosome region for 7p22 duplication syndrome. Journal of Pediatric Genetics, 4(1), 34–37. 10.1055/s-0035-1554980 27617114 PMC4906420

[mgg31829-bib-0009] Di Bartolo, D. L. , El Naggar, M. , Owen, R. , Sahoo, T. , Gilbert, F. , Pulijaal, V. R. , & Mathew, S. (2012). Characterization of a complex rearrangement involving duplication and deletion of 9p in an infant with craniofacial dysmorphism and cardiac anomalies. Molecular Cytogenetics, 5, 31. 10.1186/1755-8166-5-31 22768875 PMC3419606

[mgg31829-bib-0010] Faas, B. H. , de Leeuw, N. , Mieloo, H. , Bruinenberg, J. , & de Vries, B. B. (2007). Further refinement of the candidate region for monosomy 9p syndrome. American Journal of Medical Genetics Part A, 143A, 2353–2356. 10.1002/ajmg.a.31961 17853473

[mgg31829-bib-0011] Goitia, V. , Oquendo, M. , & Stratton, R. (2015). Case of 7p22.1 microduplication detected by whole genome microarray (REVEAL) in workup of child diagnosed with autism. Case Reports in Genetics, 2015, 1–6. 10.1155/2015/212436 PMC4393924 25893121

[mgg31829-bib-0012] Guioli, S. , Schmitt, K. , Critcher, R. , Bouzyk, M. , Spurr, N. K. , Ogata, T. , Hoo, J. J. , Pinsky, L. , Gimelli, G. , Pasztor, L. , & Goodfellow, P. N. (1998). Molecular analysis of 9p deletions associated with XY Sex reversal: refining the localization of a sex‐determining gene to the tip of the chromosome. American Journal of Human Genetics, 63, 905–908. 10.1086/302017 9718347 PMC1377405

[mgg31829-bib-0013] Güneş, S. , Ekinci, O. , Ekinci, N. , & Toros, F. (2017). Coexistence of 9p deletion syndrome and autism spectrum disorder. Journal of Autism and Developmental Disorders, 47, 520–521. 10.1007/s10803-016-2943 27878741

[mgg31829-bib-0015] Hauge, X. , Raca, G. , Cooper, S. , May, K. , Spiro, R. , Adam, M. , & Martin, C. L. (2008). Detailed characterization of, and clinical correlations in, 10 patients with distal deletions of chromosome 9p. Genetics in Medicine, 10, 599–611. 10.1097/GIM.0b013e31817e2bde 18641517 PMC2953383

[mgg31829-bib-0016] Hou, Q. , Wu, D. , Chu, Y. , & Liao, S. (2016). Clinical findings and molecular cytogenetic study of de novo pure chromosome 9p deletion: Pre‐ and postnatal diagnosis. Taiwanese Journal of Obstetrics & Gynecology, 55, 867–870. 10.1016/j.tjog.2016.11.001 28040136

[mgg31829-bib-0017] Huret, J. L. , Leonard, C. , Forestier, B. , Rethore, M. O. , & Lejeune, J. (1988). Eleven new cases of del(9p) and features from 80 cases. Journal of Medical Genetics, 25, 741–749. 10.1136/jmg.25.11.741 3070043 PMC1051577

[mgg31829-bib-0018] ISCN (2016). An international system for human cytogenetic nomenclature. McGowan‐Jordan J, Simons A, Schmid M, (eds) Karger, Basel. 10.1159/isbn.978-3-318-05979-3

[mgg31829-bib-0019] Kawara, H. , Yamamoto, T. , Harada, N. , Yoshiura, K. , Niikawa, N. , Nishimura, A. , & Matsumoto, N. (2006). Narrowing candidate region for monosomy 9p syndrome to a 4.7‐Mb segment at 9p22.2‐p23. American Journal of Medical Genetics Part A, 140A, 373–377. 10.1002/ajmg.a.31094 16419130

[mgg31829-bib-0020] Kim, E. J. , Chung, S. , Park, T. S. , & Choi, Y. (2017). A case of partial short arm deletion in chromosome 9 with inguinal hernia, testicular cystic lesion, and arthrogryposis multiplex congenita. Neonatal Medicine, 24, 88–91. 10.5385/nm.2017.24.2.88

[mgg31829-bib-0021] Koslow, E. , Reeves, P. T. , Anchan, J. , & Rohena, L. (2018). Novel case of 9p‐ deletion in a patient with cardiac pathology and a review of the literature. Journal of Clinical Research and Medicine, 1, 1–3.

[mgg31829-bib-0022] Lai, C. S. L. , Fisher, S. E. , Hurst, J. A. , Vargha‐Khadem, F. , & Monaco, A. P. (2001). A forkhead‐domain gene is mutated in a severe speech and language disorder. Nature, 413(6855), 519–523. 10.1038/35097076 11586359

[mgg31829-bib-0023] Mitsui, N. , Shimizu, K. , Nishimoto, H. , Mochizuki, H. , Iida, M. , & Ohashi, H. (2013). Patient with terminal 9 Mb deletion of chromosome 9p: Refining the critical region for 9p monosomy syndrome with trigonocephaly. Congenital Anomalies, 53, 49–53. 10.1111/j.1741-4520.2012.00362 23480358

[mgg31829-bib-0024] Ogata, T. , Muroya, K. , Ohashi, H. , Mochizuki, H. , Hasegawa, T. , & Kaji, M. (2001). Female gonadal development in XX patients with distal 9p monosomy. European Journal of Endocrinology, 145, 613–617. 10.1530/eje.0.1450613 11720880

[mgg31829-bib-0025] Onesimo, R. , Orteschi, D. , Scalzone, M. , Rossodivita, A. , Nanni, L. , Zannoni, G. F. , Marrocco, G. , Battaglia, D. , Fundarò, C. , & Neri, G. (2012). Chromosome 9p deletion syndrome and sex reversal: Novel findings and redefinition of the critically deleted regions. American Journal of Medical Genetics Part A, 158A, 2266–2271. 10.1002/ajmg.a.35489 22821627

[mgg31829-bib-0026] Ottolenghi, C. , Veitia, R. , Quintana‐Murci, L. , Torchard, D. , Scapoli, L. , Souleyreau‐Therville, N. , Beckmann, J. , Fellous, M. , & McElreavey, K. (2000). The region on 9p associated with 46, XY sex reversal contains several transcripts expressed in the urogenital system and a novel doublesex‐related domain. Genomics, 64, 170–178. 10.1006/geno.2000.6121 10729223

[mgg31829-bib-0027] Papadopoulou, E. , Sifakis, S. , Sarri, C. , Gyftodimou, J. , Liehr, T. , Mrasek, K. , Kalmanti, M. , & Petersen, M. B. (2006). A report of pure 7p duplication syndrome and review of the literature. American Journal of Medical Genetics, 140A, 2802–2806. 10.1002/ajmg.a.31538 17103460

[mgg31829-bib-0028] Pierpont, M. E. , Brueckner, M. , Chung, W. K. , Garg, V. , Lacro, R. V. , McGuire, A. L. , & Russell, M. W. (2018). Genetic basis for congenital heart disease: Revisited: A scientific statement from the American Heart Association. Circulation, 138, 606. 10.1161/CIR.0000000000000606 PMC6555769 30571578

[mgg31829-bib-0029] Quinonez, S. C. , Park, J. M. , Rabah, R. , Owens, K. M. , Yashar, B. M. , Glover, T. W. , & Keegan, C. E. (2013). 9p partial monosomy and disorders of sex development: Review and postulation of a pathogenetic mechanism. American Journal of Medical Genetics Part A, 161A, 1882–1896. 10.1002/ajmg.a.36018 23824832

[mgg31829-bib-0030] Recalcati, M. P. , Bellini, M. , Norsa, L. , Ballarati, L. , Caselli, R. , Russo, S. , Larizza, L. , & Giardino, D. (2012). Complex rearrangement involving 9p deletion and duplication in a syndromic patient: Genotype/phenotype correlation and review of the literature. Gene, 502, 40–45. 10.1016/j.gene.2012.04.030 22537675

[mgg31829-bib-0031] Ruusala, A. , & Aspenstrom, P. (2004). Isolation and characterisation of DOCK8, a member of the DOCK180‐related regulators of cell morphology. FEBS Letters, 572, 159–166. 10.1016/j.febslet.2004.06.095 15304341

[mgg31829-bib-0032] Schanze, I. , Bunt, J. , Lim, J. W. C. , Schanze, D. , Dean, R. J. , Alders, M. , Blanchet, P. , Attié‐Bitach, T. , Berland, S. , Boogert, S. , Boppudi, S. , Bridges, C. J. , Cho, M. T. , Dobyns, W. B. , Donnai, D. , Douglas, J. , Earl, D. L. , Edwards, T. J. , Faivre, L. , … Richards, L. J. (2018). NFIB haploinsufficiency is associated with intellectual disability and macrocephaly. American Journal of Human Genetics, 103, 752–768. 10.1016/j.ajhg.2018.10.006 30388402 PMC6218805

[mgg31829-bib-0033] Shimojima, K. , & Yamamoto, T. (2009). Investigation of the candidate region for trigonocephaly in a patient with monosomy 9p syndrome using array‐ CGH. American Journal of Medical Genetics Part A, 149A, 1076–1080. 10.1002/ajmg.a.32783 19396833

[mgg31829-bib-0034] Spazzapan, P. , Arnaud, E. , Baujat, G. , Nizon, M. , Malan, V. , Brunelle, F. , & Di Rocco, F. (2016). Clinical and neuroradiological features of the 9p deletion syndrome. Childs Nervous System, 32, 327–335. 10.1007/s00381-015-2957-2 26597681

[mgg31829-bib-0035] Swinkels, M. E. M. , Simons, A. , Smeets, D. F. , Vissers, L. E. , Veltman, J. A. , Pfundt, R. , de Vries, B. B. A. , Faas, B. H. W. , Schrander‐Stumpel, C. T. R. M. , McCann, E. , Sweeney, E. , May, P. , Draaisma, J. M. , Knoers, N. V. , van Kessel, A. G. , & van Ravenswaaij‐Arts, C. M. A. (2008). Clinical and cytogenetic characterization of 13 Dutch patients with deletion 9p syndrome: Delineation of the critical region for a consensus phenotype. American Journal of Medical Genetics Part A, 146A, 1430–1438. 10.1002/ajmg.a.32310 18452192

[mgg31829-bib-0036] Vinci, G. , Chantot‐Bastaraud, S. , El Houate, B. , Lortat‐Jacob, S. , Brauner, R. , & McElreavey, K. (2007). Association of deletion 9p, 46, XY gonadal dysgenesis and autistic spectrum disorder. Molecular Human Reproduction, 13, 685–689. 10.1093/molehr/gam045 17644778

[mgg31829-bib-0037] Wagstaff, J. , & Hemann, M. (1995). A familial ‘‘balanced’’ 3;9 translocation with cryptic 8q insertion leading to deletion and duplication of 9p23 loci in siblings. American Journal of Human Genetics, 56, 302–309. 7825591 PMC1801320

[mgg31829-bib-0038] Yang, Y. , Wang, C. , Wang, F. , Zhu, L. , Liu, H. , & He, X. (2012). Novel chromosomal translocation t(11;9)(p15;p23) involving deletion and duplication of 9p in a girl associated with autism and mental retardation. Gene, 502, 154–158. 10.1016/j.gene.2012.04.036 22555022

